# Effects of Polyethylene Terephthalate Particle Size on the Performance of Engineered Cementitious Composites

**DOI:** 10.3390/polym16152143

**Published:** 2024-07-28

**Authors:** Shijia Chen, Runan Liu, Liuyi Liu, Xinying Huang, Jiaxiang Lin

**Affiliations:** School of Civil and Transportation Engineering, Guangdong University of Technology, Guangzhou 510006, China; 3121002695@mail2.gdut.edu.cn (S.C.); 2112209125@mail2.gdut.edu.cn (R.L.); 661liuly@gmail.com (L.L.); 3121003063@mail2.gdut.edu.cn (X.H.)

**Keywords:** polyethylene terephthalate (PET) aggregate, particle size, engineered cementitious composite (ECC), interfacial transition zone (ITZ), mechanical properties

## Abstract

This study utilizes polyethylene terephthalate (PET) aggregate of different particle sizes (21 μm, 107 μm, and 244 μm) to replace natural aggregate in the preparation of PET-modified engineered cementitious composite (P-ECC). The impact of PET aggregate particle size on the performance of P-ECC is examined herein from micro to macro levels. The focus is on the influence patterns and mechanisms of P-ECC’s workability, its basic mechanical properties, and its microstructure. Crack parameters are processed to quantitatively analyze crack development patterns. Using microscopic techniques, the interfacial transition zone (ITZ) between different aggregates and the cement matrix is compared, and the failure mechanism of P-ECC is analyzed. The results show that the incorporation of PET aggregate can improve P-ECC’s workability and reduce its self-weight, but incorporation has a negative effect on compressive strength. Additionally, the particle size of PET aggregate significantly affects the uniaxial tensile performance of P-ECC. Compared to conventional ECC, the tensile strength of P-S (21 μm PET) increased the most markedly (18.1%), and the ultimate tensile strain of P-M (107 μm PET) increased the most markedly (66.0%), with both demonstrating good crack control and deformation energy dissipation capabilities. The uniaxial tensile performance of P-L (244 μm PET) was lower than that of the conventional ECC. Microscopic tests revealed that the increase in PET aggregate particle size enlarges the ITZ width and its surrounding pores. Appropriate pore enlargement is beneficial for enhancing tensile ductility, while excessive pores have a negative effect. The study results reveal the impact of PET aggregate particle size on the performance of P-ECC, providing new insights for the performance optimization of ECC.

## 1. Introduction

Engineered cementitious composite (ECC) is a high-performance fiber-reinforced cementitious composite that was designed based on micromechanics theory [1,2,3]. The incorporation of 1–2 vol.% of synthetic fibers into ECC enhances its tensile deformation capacity by 200–300 times and significantly increases its energy absorption capability compared to conventional concrete [4,5]. The tensile stress–strain curve of ECC resembles that of metallic materials more than conventional concrete [6]. Hence, ECC is also known as strain-hardening cementitious composite (SHCC) and is characterized by pseudo strain-hardening behavior, ultra-high tensile ductility, and excellent multiple cracking phenomena. These features have made ECC a research hotspot both domestically and internationally for a long time [7,8,9,10,11]. The material advantages of ECC give it broad application prospects in engineering construction, significantly enhancing concrete’s structural safety and durability to meet the demand for high-performance materials in modern engineering.

Given these higher engineering requirements and more complex service environments, there is still significant room for improvement in the various properties of ECC [12,13,14,15,16,17]. Many researchers have focused on fibers to optimize ECC performance [18,19,20,21]. However, ECC preparation, based on micromechanical models, is highly dependent on natural fine aggregates [21,22]. Thus, investigating the modification of ECC using new artificial aggregates as substitutes for natural aggregates is a promising research direction. In ordinary concrete, using artificial aggregates introduces defects, which reduce the concrete’s strength and elastic modulus [23,24]. However, small defects can effectively improve the tensile ductility of ECC. Chen et al. [25] developed SC-ECC by using calcined waste marine clay as a natural aggregate substitute, which increased the ECC’s ultimate tensile strain by 204.8%. Zhou et al. [26] developed high-performance green lightweight ECC using recycled fine aggregate and found that the inherent defects in this aggregate could effectively reduce ECC density and increase its ultimate tensile strain. Additionally, the particle size of aggregates significantly affects the mechanical properties of ECC [27]. Xia et al. [28] prepared ECC using various aggregates and found that coarse standard sand reduced tensile deformation performance, while large-particle-sized river sand adversely affected fiber dispersion. Conversely, Guan et al. [29] showed that using large-particle-sized and high-volume river sand in ECC maintained ultra-high tensile strain performance despite a slight reduction in tensile strength, easily satisfying the PSH criterion. Qiu et al. [30] found that incorporating expanded perlite (EPP) as an aggregate into ECC significantly decreased the compressive strength as the EPP particle size increased. Therefore, research on recycled aggregates and aggregate particle sizes is crucial for the further development of ECC.

In recent years, the amount of plastic waste has surged, with plastic bottles made of polyethylene terephthalate (PET) being a major component [31,32,33]. It is estimated that nearly 60% of PET plastic waste is directly landfilled or discarded, posing a serious threat to the ecosystem [34,35]. PET has extremely low biodegradability [36,37], and traditional disposal methods like landfilling and incineration cause severe environmental pollution [38,39]. Some scholars have suggested recycling large amounts of PET waste in the construction field [40,41,42,43]. However, most studies have focused on using PET as fibers in concrete, which recycles very little PET [40,44]. Meanwhile, the rapid increase in concrete production has dramatically raised the demand for natural aggregates [45,46,47,48,49]. The shortage of river sand has led to significant price increases, severely impacting the construction industry [50,51,52]. Thus, several scientific institutions propose using recycled aggregates as substitutes for natural aggregates [45,47]. Addressing PET waste disposal issues by using PET as an aggregate in concrete is a potential solution to the shortage of natural aggregates. Some researchers have studied the use of recycled PET aggregates in ordinary concrete production [53,54,55]. Marzouk et al. [56] found that PET aggregate has a particle size similar to quartz sand and can be successfully used as a concrete aggregate substitute. Frigione et al. [44] indicated that PET aggregate, as a natural aggregate substitute, could increase concrete toughness and provide some ductility. Kangavar et al. [45] showed that incorporating a small amount of PET aggregate could effectively reduce concrete density while ensuring strength. Fakhruddin et al. [57] found that PET aggregate could significantly enhance the crack resistance of concrete. Thus, PET aggregate positively affects concrete density, crack control capability, and ductility. However, most studies have focused on PET aggregate replacement rates in ordinary concrete, with limited research on modifying ECC with PET aggregate. Therefore, investigating the influence patterns and mechanisms of different PET aggregate particle sizes on ECC performance is crucial for further optimizing this new composite material, with significant practical implications. Based on this finding, the present study explores an innovative material performance optimization approach: using PET aggregate to replace natural aggregate in ECC. Compared to traditional aggregates, PET aggregates have superior plasticity and lower elastic modulus, further enhancing the tensile ductility of ECC. This innovative method not only improves the mechanical properties of material but also positively impacts environmental protection and resource utilization.

This study prepares engineered cementitious composites modified with PET aggregate (P-ECC) of different particle sizes and investigates the various properties of P-ECC under different PET aggregate particle sizes from macro to micro levels, including workability, density, compressive strength, uniaxial tensile performance, and microstructure. The focus is on the influence patterns and mechanisms of PET aggregate particle size on ECC’s multiple cracking behavior, axial tensile performance, and microstructure. These explorations help understand the failure mechanism of P-ECC and the bonding effect of the ITZ between PET aggregate and the cement matrix, providing reference value for the practical engineering application of P-ECC.

## 2. Experimental Program

### 2.1. Materials and Mix Proportion

**Figure 1:**
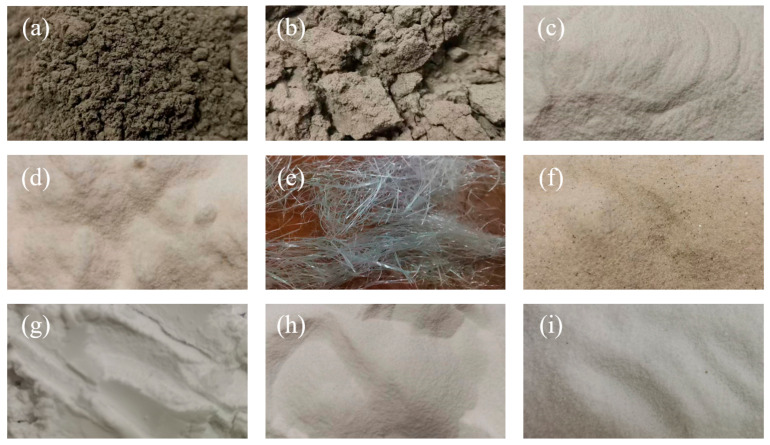
Raw materials: (**a**) cement, (**b**) FA, (**c**) DF, (**d**) VMA, (**e**) PE fiber, (**f**) QP, (**g**) PET-S, (**h**) PET-M, and (**i**) PET-L.

**Figure 2:**
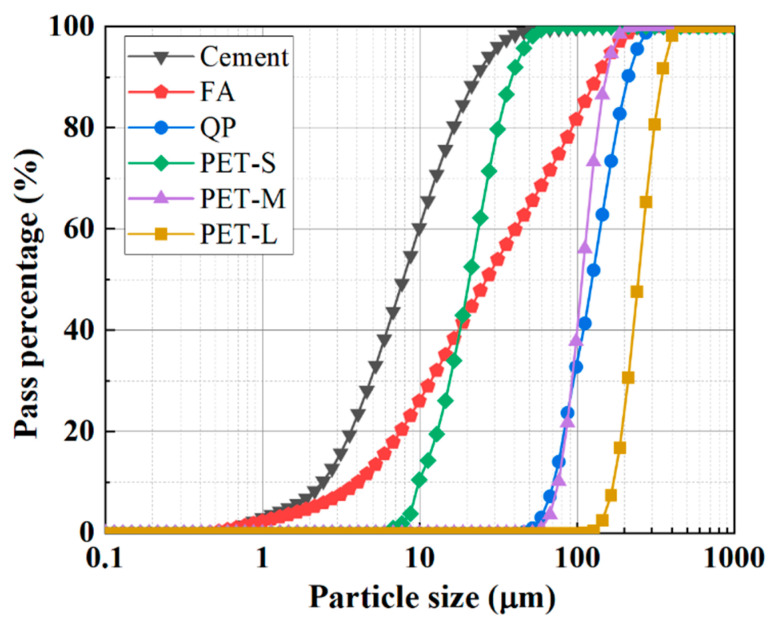
Particle size distribution of the raw materials.

Figure 1 and Figure 2 display the raw materials and their particle size distribution for P-ECC. The raw materials consist of water, cementitious materials, aggregates, fibers, and admixtures. The cementitious materials include P II. 52.5R ordinary Portland cement (Guangzhou Heidelberg Yuexiu Cement Co., Ltd., Guangzhou, China) and fly ash (FA, Longze Water Purification Materials Co., Ltd., Gongyi, China), with Table 1 showing the chemical composition of the cementitious materials. The aggregates include quartz powder (QP, Jiangmen Xiangyuan Co., Ltd., Guangzhou, China) and polyethylene terephthalate plastic powder (PET, DuPont Co., Ltd., Wilmington, CA, USA). The admixtures include a viscosity-modifying agent (VMA, Shandong Xinfuman Chemical Technology Co., Ltd., Zibo, China), a defoamer (DF, Nanjing Yaojie Energy Saving Technology Co., Ltd., Nanjing, China), and a high-range water-reducing agent (HRWRA, BASF Co., Ltd., Berlin, Germany). The main component of VAM is carboxypropyl methyl cellulose, with a purity of 90%. DF is a mixture of liquid hydrocarbons, polyethylene glycol, and amorphous silica, with an active ingredient content of 65%. The solid content of HRWRA is 49–51%, with a pH value of 5.0–8.0. Polyethylene fiber (PE fiber, Beijing Quantum Terra New Materials Technology Co., Ltd., Beijing, China) was used, with its physical properties detailed in Table 2. To study the impact of different PET aggregate particle sizes on P-ECC performance, three different particle sizes (PET-S, PET-M, PET-L) of PET aggregates were selected. PET-M was chosen to match the particle size distribution of quartz powder for the replacement experiment [58]. Table 3 compares the physical properties of QP and PET aggregates.

The mix proportions for this experiment were determined by referencing previous related studies and by conducting multiple trial mixing experiments. Four groups of P-ECC specimens were set up for this experiment: one control group containing only QP and three groups with different PET aggregate particle sizes, with a PET aggregate volume replacement rate of 15%. The groups with PET aggregates were named P-S, P-M, and P-L, in order of increasing particle size. Based on existing studies [45,59,60], at a 15 vol.% PET aggregate replacement rate, concrete shows greater deflection under bending loads, demonstrating excellent deformation capacity while maintaining a high level of strength performance, and improving the workability of fresh paste. To ensure uniform dispersion of the PE fibers and good workability of the paste, the optimal mix proportions were determined through multiple trials, as shown in Table 4.

### 2.2. Mixing Process

**Figure 3:**
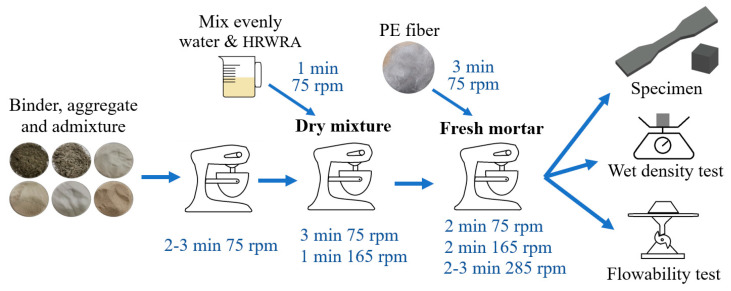
Mixing process.

The mixing process of P-ECC is shown in Figure 3. The specimens were prepared using a 30-L planetary mortar mixer with 3 speed settings (low speed 75 r/min, medium speed 165 r/min, and high speed 285 r/min). First, the cementitious materials, aggregates, thickening agent, and defoamer were added to the mixer and mixed at low speed to ensure that the dry materials were evenly blended. Next, pre-mixed water and water-reducing agent were added. Once the mixture had fully absorbed the water, it was mixed at medium speed until a uniform paste was achieved. Finally, the mixer was returned to low speed, and the PE fibers were gradually added, adjusting to medium and high speeds based on the dispersion of the fibers to ensure that they were fully distributed in the paste.

After mixing, the fresh paste was used for molding, flowability testing, and wet density testing. During molding, the molds were placed on a vibration table to minimize air bubbles inside the specimens. The mold surfaces were covered with a thin film to prevent moisture evaporation. The specimens were demolded after curing at room temperature for 24 h, then placed in water for 28 days before being taken out for testing.

## 3. Experimental Setup and Procedure

### 3.1. Flowability Test

The flowability of the different fresh paste mixtures was tested according to ASTM C1437-20 [61]. The standard test mold is frustum-shaped, with an upper diameter of 70 mm, a lower diameter of 100 mm, and a depth of 50 mm. The flowability test includes the following steps: first, wet the test mold, then fill it with the mixture and compact it. After vertically removing the mold, immediately start the test machine. After completing the test according to the standard, measure two orthogonal diameters of the paste base with a caliper and take their average value *D* to quantify the flowability of the paste. The calculation formula is shown in Equation (1).

(1) $$Flow=\frac{D-100\;mm}{100\;mm}\times 100\%$$

### 3.2. Density Test

The wet density and dry density of each group of specimens were tested according to ASTM C138/C138M-23 [62]. A 5-L cylindrical container made of steel was used for the experiment. Before testing, the container should be dried and weighed to obtain its tare weight. Then, the mixed concrete is poured into the container, vibrated, and the surface is leveled. The weight is measured to calculate the wet density. After curing, the specimens are dried until their weight stabilizes, and the weight is measured again to calculate the dry density.

### 3.3. Compression Test

According to ASTM C109/C109M-21 [63], the compressive strength test was conducted using 50 mm × 50 mm × 50 mm cubes. In this experiment, a YAW-5000 computer-controlled electro-hydraulic servo pressure testing machine was used to load the cubes. The loading was performed in a stress-controlled mode with a loading rate of 0.72 MPa/s.

### 3.4. Tensile Test

According to JSCE [64], the axial tensile test used dog-bone-shaped specimens with dimensions of 330 mm × 60 mm × 13 mm. The loading mode was displacement-controlled, with a loading rate of 0.5 mm/min. Two linear variable displacement transducers (LVDT) were installed in the 80-mm measuring section at the center of the specimen to measure tensile deformation, and data were collected in combination with a stress sensor. Additionally, to prevent eccentric loading, universal joints were installed at both ends of the fixture.

This study particularly focused on multiple cracking behavior under tensile load. Therefore, during the test, a high-definition camera was fixed on a tripod, and images of the measuring section were taken at a frequency of 0.2 Hz. The characteristic images were processed using image binarization. Furthermore, to facilitate the subsequent observation of cracks and image processing, the surface of the specimen should be pre-coated with white paint.

### 3.5. SEM and EDS

After the tensile test, samples with dimensions not exceeding 5 mm were prepared for scanning electron microscopy (SEM) and energy-dispersive spectroscopy (EDS) tests. First, SEM was used to capture micrographs of the aggregate raw materials, comparing the shape and surface smoothness of different aggregates. Second, EDS was employed to identify the locations and chemical compositions of different aggregates, allowing for a comparison of the widths of the interfacial transition zones (ITZ) between different aggregates and the cement matrix. Finally, SEM was used to capture and analyze the microstructures of P-ECC samples from each mix proportion.

## 4. Results and Discussion

### 4.1. Flowability

**Figure 4:**
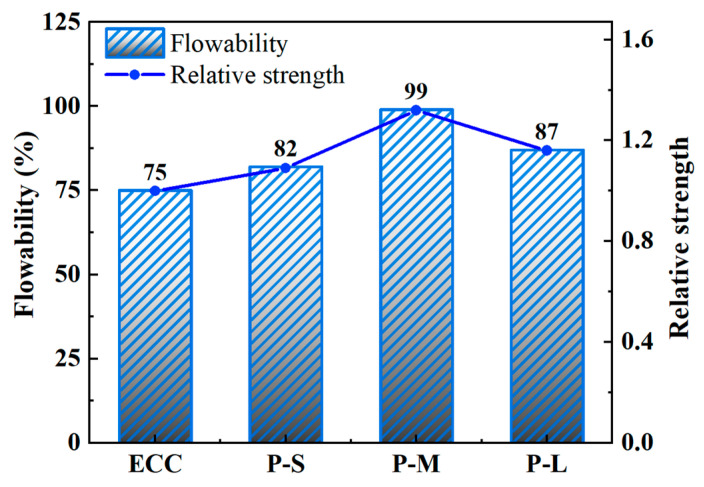
The flowability of the various P-ECC samples.

Figure 4 shows the flowability of fresh mixes for each mix proportion. Changes in flowability significantly affect the fiber dispersion and workability of ECC, directly impacting its pseudo-strain hardening behavior and effective fiber utilization [65]. From Figure 4, it is evident that compared to ordinary ECC, the flowability of P-ECC is generally improved with the addition of PET aggregates. This is because PET materials are hydrophobic, absorbing less water during mixing than natural aggregates [66], which increases the water–cement ratio and improves the flowability of the slurry.

Simultaneously, with an increase in PET aggregate size, the flowability of ECC shows an initial increase, followed by a decreasing trend. Among them, P-M exhibits the highest flowability, reaching 99%. For P-S, reducing the size of PET aggregates increases the total surface area of the aggregates, leading to higher water absorption rates and a lower water–cement ratio in the slurry. Additionally, the use of ultra-light plastic aggregates may cause uneven distribution and segregation of the mixture [67], potentially leading to fiber clustering and negatively affecting slurry flowability. For P-L, using large-sized PET aggregates instead of quartz powder reduces aggregate packing density and increases inter-aggregate voids. Consequently, more cementitious material is needed to fill these voids, resulting in decreased flowability. This is consistent with the findings of Saikia et al. [39]. Therefore, replacing natural aggregates with PET-M is more advantageous for enhancing the flowability of fresh P-ECC mixes, optimizing their workability and fiber dispersion, and further strengthening the pseudo-strain hardening behavior of ECC.

### 4.2. Density

**Figure 5:**
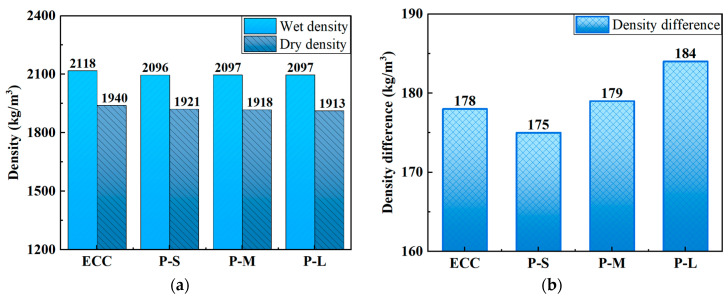
The density of P-ECC: (**a**) wet density and dry density, (**b**) density difference.

Table 5 and Figure 5 present the wet density, dry density, and their differences for each mix proportion. According to standard JGJ/T12-2019 [68], the dry density of lightweight concrete should not exceed 1950 kg/m^3^, and the ECC specimens in this study meet this requirement. As shown in Table 5, compared to ordinary ECC, both the wet and dry densities of P-ECC are lower. This can be attributed to the lower density of PET aggregates compared to quartz powder, which aligns with previous research conclusions [60,66,69]. Figure 5 shows that with an increase in PET aggregate size, the dry density of P-ECC decreases, and the difference between wet and dry densities increases. This indicates that larger PET aggregate sizes lead to higher saturated moisture content in ECC, further implying an increase in the volume of pores in ECC. While a decrease in density in conventional concrete indicates material defects that are detrimental to tensile performance, in ECC, an appropriate level of defects not only reduces the self-weight but also enhances its multiple cracking behavior, thereby improving tensile strain capacity.

### 4.3. Compressive Behavior

#### 4.3.1. Failure Mode

**Figure 6:**
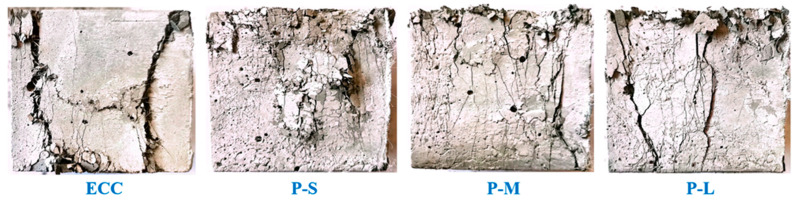
The typical compressive failure morphology of the various P-ECC samples.

Figure 6 shows the typical failure modes of axial compression specimens for each group. Compared to ordinary ECC, the addition of PET-M changes the failure mode from fewer but wider cracks to more numerous and finer cracks. This is because PET aggregates introduce tiny defects into ECC, which then develop many small cracks during compression, thereby slowing down the expansion of existing cracks.

As shown in Figure 6, with increasing PET aggregate size, the number of cracks in P-ECC gradually decreases. In contrast to P-M, P-S specimens exhibit more blocky fragments on the surface. This is due to the smaller and more numerous PET-S aggregates, which are more uniformly dispersed in the concrete and effectively disperse the stress on the cubic specimens. At the same time, smaller aggregate sizes increase the interfacial transition zone (ITZ) area, resulting in many intersecting fine cracks on the concrete’s surface. When the load level increases, these cracks further extend and connect to form smaller blocky fragments. In contrast, cracks in P-L specimens are wider and more concentrated. This is because the introduction of larger aggregate sizes increases the number of large voids or defects in the concrete structure. Under compressive stress, these locations experience stress concentration, leading to the rapid development of wider cracks. The appearance of wider cracks further enhances stress concentration, thereby further expanding cracks at these locations, leading to a relatively concentrated crack development position. Therefore, the introduction of PET aggregate sizes should not be too large; otherwise, the ECC may still exhibit excessively wide crack failure modes.

#### 4.3.2. Compressive Strength

**Figure 7:**
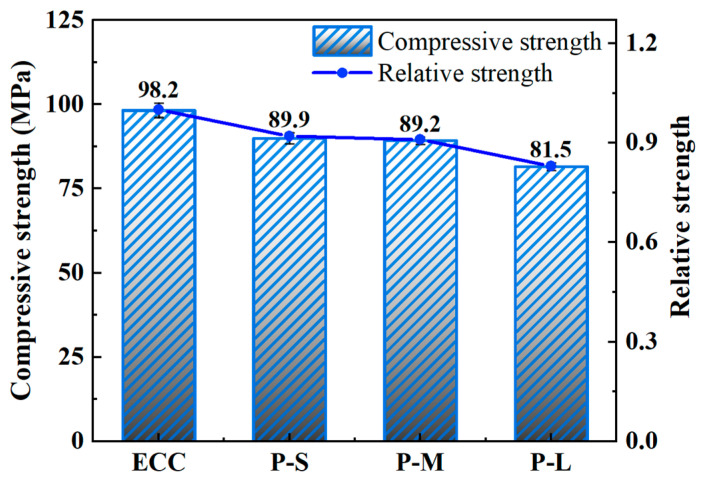
The compressive strength of the various P-ECC samples.

Table 6 and Figure 7 present the compressive strength of cube specimens with different mix proportions. The results in the table represent the average values of three specimens, with a standard deviation not exceeding 2.0 MPa. From Figure 7, it can be observed that the addition of PET aggregates reduces the compressive strength of ECC, and the size of the aggregates also influences the compressive strength of ECC. Specifically, the compressive strength difference between P-S and P-M is relatively small. PET-S aggregates have a larger surface area, leading to greater contact with the cement matrix. This extensive contact affects the bonding between the cement matrix and the aggregates, reducing the compressive strength of P-S. P-M has a lower density and larger pores, which also results in decreased compressive strength. Therefore, the difference in compressive strength between P-S and P-M is relatively small. In contrast, when replacing aggregates from PET-M to PET-L, the compressive strength decreases by 8.63%. This indicates that when the particle size of introduced PET aggregates reaches a certain size, it has a more pronounced negative effect on the compressive strength of the resulting material. From the perspective of the compressive strength mechanism of ECC, after matrix cracking, the PE fibers provide bridging capability. When the cracks reach a certain width, the PE fibers lose their bridging ability, leading to ECC failure. The wider cracks in P-L specimens, combined with the significantly lower elastic modulus of PET aggregates compared to quartz powder, cause PE fibers to lose this bridging ability rapidly, resulting in a significant reduction in compressive strength. Therefore, adjusting the particle size of PET aggregates can achieve superior compressive performance in ECC.

### 4.4. Tensile Behavior

#### 4.4.1. Failure Mode

**Figure 8:**
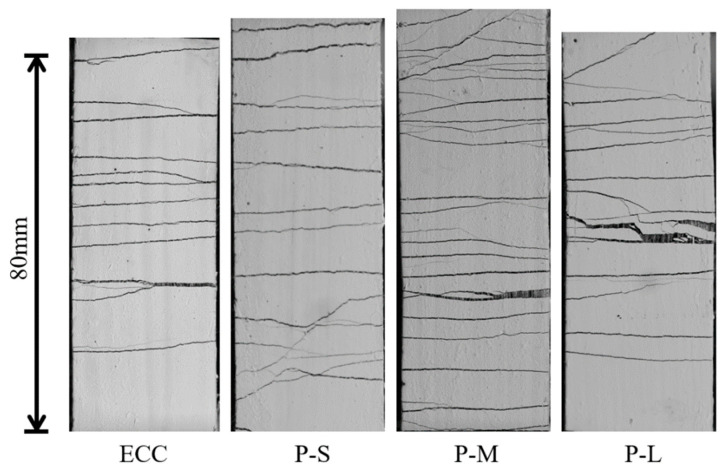
The typical tensile failure morphology of the P-ECC.

**Figure 9:**
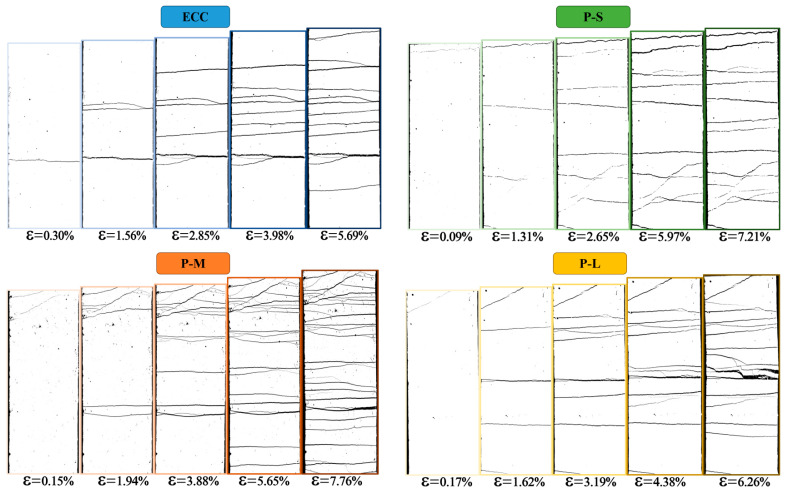
The process of crack evolution in the P-ECC samples.

To observe the initiation and development of cracks during the tensile process, image binarization methods were employed to process segment images of the specimens under test, yielding crack distribution images at different tensile strains. Figure 8 and Figure 9 depict the typical failure modes and crack evolution process of P-ECC under tensile loading. It can be observed that all mix proportions of ECC in this study exhibited a ductile failure mode characterized by multiple crack initiation and propagation, with the trend in the number of cracks generally following the variation in tensile strain capacity. With the addition of PET aggregates, the number of cracks in the P-M sample significantly increased, somewhat alleviating the failure of the material. In contrast, the P-S material, which used smaller aggregates, was denser and more uniform, resulting in fewer additional cracks. Conversely, P-L, which used larger aggregates, exhibited wider cracks due to larger voids, and the number of cracks almost did not increase. Figure 9 illustrates that after applying the load, the specimen undergoes a brief period of elastic deformation. When the tensile stress at the weakest point exceeds the tensile strength that the matrix can bear, cracks initiate at that location, with PE fibers providing bridging. As the tensile load increases, the number of cracks continues to increase, and existing cracks expand. When cracks reach a certain width, the bridging effect of PE fibers significantly weakens, leading to gradual softening and failure of the specimen. Therefore, the strain capacity of ECC primarily originates from multiple cracking stages, where both the number and width of cracks are crucial factors that determine whether ECC can exhibit excellent strain capacity.

#### 4.4.2. Crack Parameters

**Figure 10:**
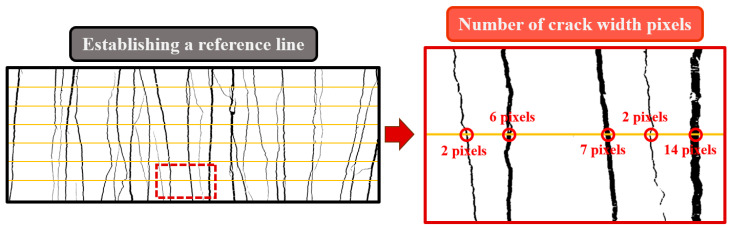
The processing process to determine the crack parameters of the P-ECC.

**Figure 11:**
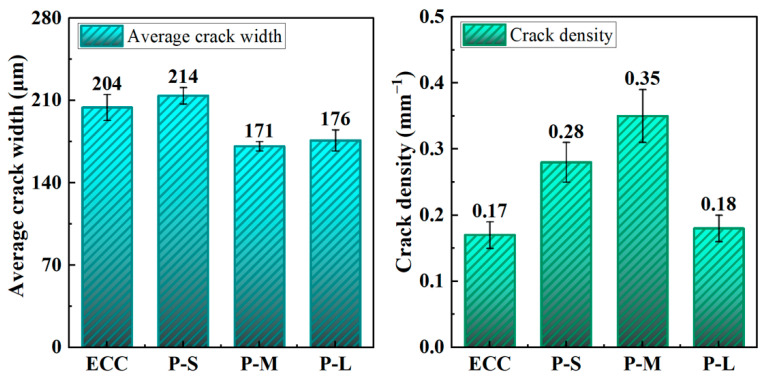
The average crack width and crack density of the P-ECC.

Figure 10 illustrates the quantitative analysis of the effect of PET aggregates on the multiple cracking behavior of ECC. Characteristic images processed through binarization were used to establish reference lines parallel to the direction of tension, counting the pixels at intersections between the cracks and reference lines to obtain specific crack parameters. Table 7 and Figure 11 present the average crack width and crack density obtained from processing various mix proportions.

From Figure 11, it is evident that replacing quartz powder with PET aggregates of different sizes leads to significant differences in crack characteristics in ECC. When using PET-M, which has a particle size close to that of quartz powder, the ECC showed a significant increase in crack density and a significant decrease in average crack width. This is due to the introduction of tiny pores by PET aggregates, which can initiate more fine cracks and, thus, slow down crack propagation. In contrast, compared to P-M, the P-S sample exhibited a reduced crack density and increased average crack width. P-S material is denser and more uniform, making it less prone to developing new cracks and more inclined to expand existing ones. Conversely, P-L has larger voids, making cracks prone to excessive expansion at large defect locations, leading to premature failure and reduced crack density, which is similar to findings by Chandrasekhar et al. [27] on coarse aggregates. Notably, compared to ordinary ECC, the average crack width in the P-M group decreased from 204 μm to 171 μm, a reduction of 16.2%, while the crack density increased from 0.17 cracks/mm to 0.35 cracks/mm, an increase of 106%. These changes represent the highest values among the mix proportions with different PET aggregate sizes, highlighting P-M’s superior crack control capability in terms of these two uniaxial tensile performance indicators. Therefore, within the scope of this study, using PET-M is more advantageous for producing denser cracks and reducing the crack width in ECC.

#### 4.4.3. Tensile Stress–Strain Curves

**Figure 12:**
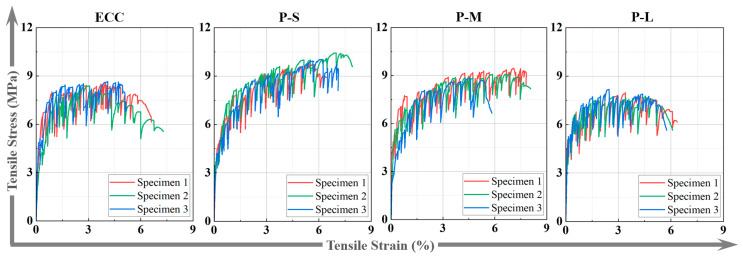
The stress–strain curves of the P-ECC.

Figure 12 shows the tensile stress–strain curves of dog-bone-shaped specimens for each group. Under axial tension, all ECC specimens exhibit typical pseudo-strain hardening behavior, which is characterized by three stages in the stress–strain curve: the elastic stage, pseudo-strain hardening stage, and softening stage. The pseudo-strain hardening stage is directly related to the ductility performance of P-ECC. From Figure 12, compared to ordinary ECC, both P-S and P-M show longer pseudo-strain hardening stages, while P-L shows less variation. This indicates that P-S and P-M exhibit more significant multiple cracking behavior and can demonstrate superior ductility performance.

#### 4.4.4. Tensile Characteristic Parameters

**Figure 13:**
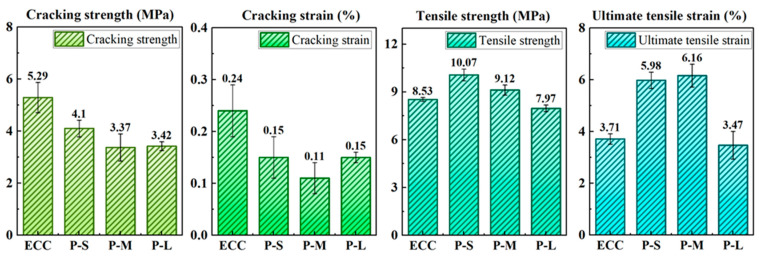
The characteristic parameters of direct tensile testing.

Table 8 and Figure 13 present the specific experimental data from axial tensile tests on P-ECC groups, including initial cracking strength, initial cracking strain, tensile strength, and ultimate tensile strain. The data represent the average values of three specimens. From Figure 13, after incorporating PET aggregates, both the initial cracking strength and initial cracking strain of ECC show varying degrees of decrease. According to Li’s research [70], this decrease in initial cracking strength is beneficial for the multiple cracking behavior of ECC. Therefore, the use of PET aggregates effectively promotes the generation of microcracks in ECC, providing a basic condition for improving ductility.

When using PET aggregates of different sizes, the tensile strength of P-ECC gradually decreases with increasing aggregate size. This is because larger PET aggregate sizes reduce the density of ECC, increase its porosity, and consequently lower its tensile strength. Additionally, compared to ordinary ECC, both P-S and P-M show significant enhancements in strain capacity, while P-L shows less variation. This enhancement may be related to the pore size in the microstructure of ECC. It is noteworthy that both the tensile strength and ultimate tensile strain of P-S and P-M are positively optimized. Tensile strength increases by a maximum of 18.1% (P-S), and ultimate tensile strain increases by a maximum of 66.0% (P-M). Based on the above analysis, within the scope of this study, using 15 vol.% of PET-S and PET-M can significantly improve the deformation capacity of P-ECC, while slightly increasing the tensile strength, which is practically meaningful for engineering applications.

#### 4.4.5. Strain Energy

**Figure 14:**
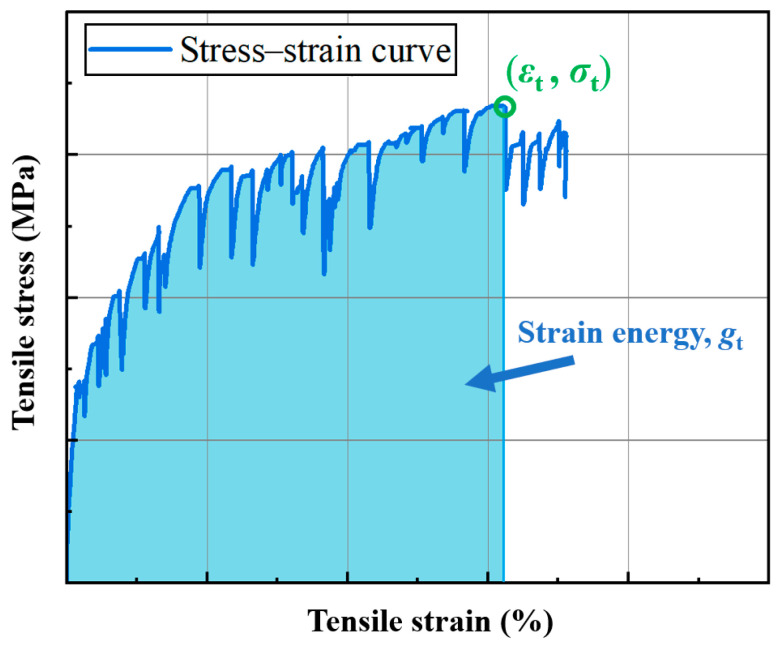
The calculation of strain energy.

**Figure 15:**
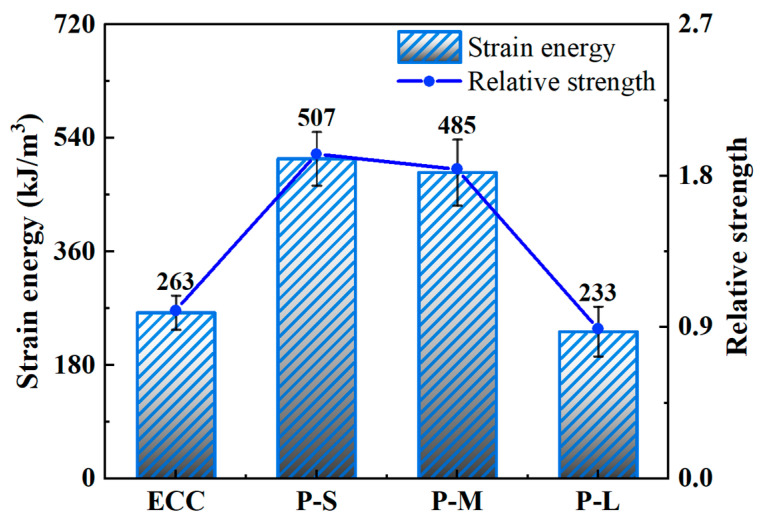
The strain energy of the P-ECC samples.

Strain energy is the energy absorbed by a specimen when forming a unit fracture surface during tension, providing a comprehensive assessment of the tensile performance of P-ECC in conjunction with tensile strength and ultimate tensile strain, as shown in Figure 14. The calculation of strain energy involves determining the area under the stress–strain curve within the integration range of [0, *ε*_t_]. Table 9 and Figure 15 present the calculated strain energy results for the various mix proportions in this study.

From Figure 15, it is evident that compared to ordinary ECC, both the P-S and P-M samples exhibit significant increases in strain energy, while P-L shows minimal change. Specifically, the P-S sample achieves the highest strain energy at 507 kJ/m^3^, marking a 92.8% increase. The increase in strain energy is primarily influenced by two factors: firstly, the P-S and P-M samples demonstrate more pronounced multiple cracking behavior, allowing more PE fibers to bridge and dissipate more energy. Secondly, as the particle size of the PET aggregates increases, the strength of the ECC decreases, resulting in less energy absorption when forming fracture surfaces. When using PET-L aggregate, the negative effects brought by artificial defects outweigh the positive impacts. This leads to a significant decrease in the tensile strength and ultimate tensile strain of P-L, resulting in a substantial reduction in strain energy. In conclusion, the inclusion of PET-S and PET-M significantly enhances the multiple cracking behavior of ECC and prolongs the multiple cracking stage, thereby improving the strain energy of ECC.

### 4.5. Microstructure Analysis

#### 4.5.1. Interface Transition Zone Characterization

**Figure 16:**
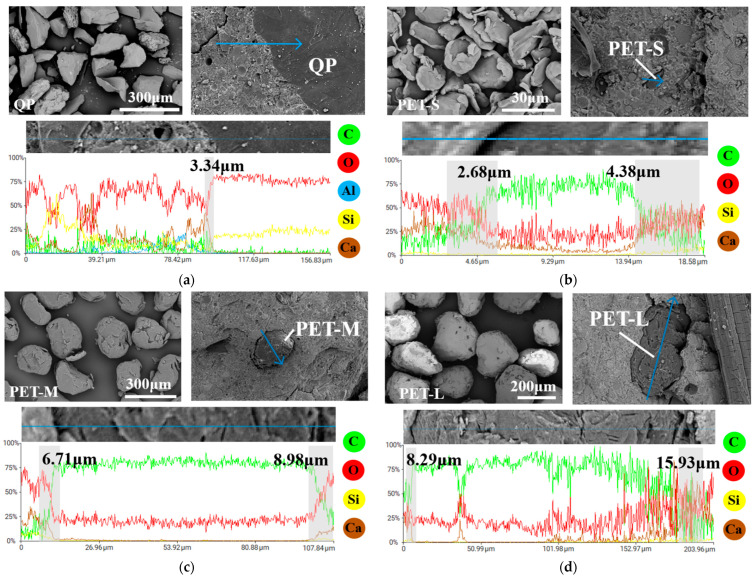
The micrographs and EDS analysis results of the QP and PET aggregates: (**a**) QP, (**b**) PET-S, (**c**) PET-M, and (**d**) PET-L.

Figure 16 shows the micrographs and EDS analysis results for quartz powder and three different sizes of PET aggregates. The EDS line scan was performed along the arrow direction, passing through the interface transition zone (ITZ) between the cement matrix and aggregates. The width of the ITZ is an important parameter, reflecting the bond strength between aggregates and the cement matrix. Proper weakening of this bond strength is advantageous for ECC by forming microcracks, promoting multiple cracking behavior, and enhancing the strain hardening characteristics.

From Figure 16, it can be observed that the ITZ width between PET aggregates and the cement matrix is greater than that between quartz powder and the cement matrix, and it widens with increasing aggregate size. This indicates that the bonding between PET aggregates and the cement matrix is weaker than that with quartz powder, and this effect becomes more pronounced with increasing PET aggregate size. From the perspective of aggregate shape, PET aggregates tend to be spherical, with fewer sharp edges and a lower modulus of elasticity compared to quartz sand, significantly reducing the interlocking friction between the PET aggregates and the cement matrix. Regarding surface smoothness, quartz sand is smooth, whereas PET aggregates have scratches from the manufacturing process, which slightly enhance friction but increase the ITZ width. In terms of aggregate size, smaller-sized PET-S aggregates are more easily filled with cementitious material, whereas larger-sized PET-L aggregates are more prone to forming voids, resulting in a wider ITZ. Additionally, due to the smaller aggregate sizes, the total area of the ITZ between the PET-S and the cement matrix increases, which also somewhat reduces the bond strength between the aggregate and the cement matrix. Therefore, compared to quartz sand, PET aggregates exhibit weaker bonding and a more pronounced ITZ with the cement matrix, which becomes more apparent with increasing PET aggregate size. By adjusting the size of the PET aggregate, the bond strength between the aggregate and the matrix can be controlled to balance the various properties of ECC.

#### 4.5.2. Microstructure

**Figure 17:**
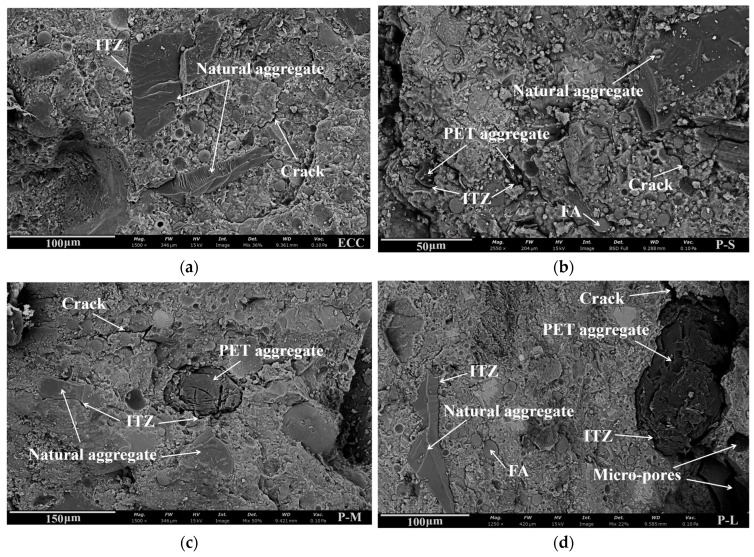
The SEM images of the microstructure of the P-ECC samples: (**a**) ECC, (**b**) P-S, (**c**) P-M, and (**d**) P-L.

Figure 17 shows scanning electron microscope (SEM) images of the different mix proportions. In comparison, samples with P-S exhibit the smallest pores, and P-M and conventional ECC show moderate density, while P-L displays larger pores. In Figure 17b, the P-S sample at high magnification shows good compactness, possibly due to its smaller particle size and greater quantity, allowing for more uniform distribution within the concrete and, thus, a denser structure. In contrast, the use of PET-L reduces the density of aggregate packing, decreasing its ability to fill small voids and resulting in wider gaps around the PET aggregates. This structural characteristic causes stress concentrations during loading, thereby reducing the material’s strength performance.

Changes in material porosity also lead to significant differences in the crack evolution behavior of ECC. When ECC has smaller pores, increasing porosity favors the appearance of microcracks, which can enhance the material’s strain capacity. However, excessive porosity leads to the over-expansion of cracks at pore locations, resulting in premature failure of the specimen. Therefore, the larger pores in the P-L samples negatively impact multiple cracking behavior, significantly reducing its ductility compared to P-M. In summary, increasing the particle size of the PET aggregates creates a more porous microstructure, which may weaken the mechanical properties of the resulting P-ECC. Therefore, it is crucial to judiciously control the particle size of PET aggregates to ensure the structural effectiveness and performance of the composite material.

### 4.6. Comprehensive Performance Analysis

**Figure 18:**
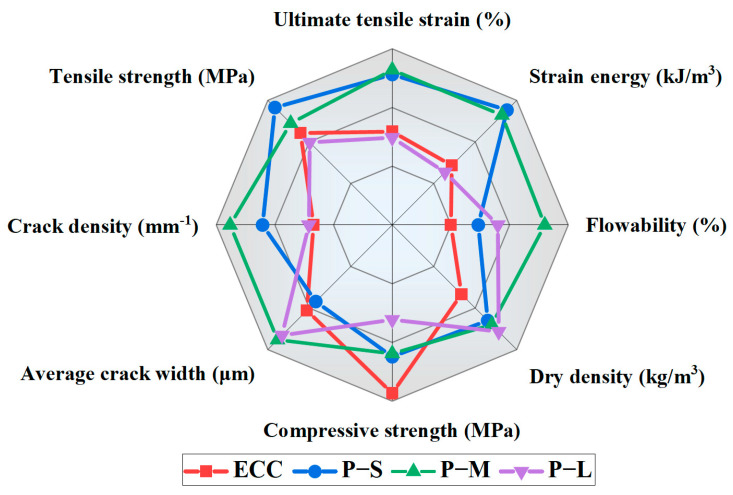
Comprehensive performance evaluation.

Figure 18 presents the comprehensive performance of each mix proportion, considering eight indicators: workability, dry density, compressive strength, average crack width, crack density, tensile strength, ultimate tensile strain, and strain energy. The axes are arranged radially from the center outward, with average crack width and dry density represented on the negative axis, and the rest on the positive axis. From the figure, it can be observed that the P-M sample exhibits the most balanced performance indicators, with the largest enveloping area, followed by P-S. Conventional ECC shows prominent compressive strength, while the P-L sample demonstrates better average crack width and dry density indicators. Additionally, both P-S and P-M show excellent ductility. In terms of tensile performance, P-S shows the best tensile strength and energy dissipation capability. From a crack control perspective, P-M exhibits the best average crack width and crack density. Therefore, in practical engineering applications, P-S can be chosen as the aggregate when emphasizing the ECC’s strength performance, while P-M is preferable when prioritizing the crack control capabilities of the material. Despite the negative impact of large PET aggregates on the produced composite, this study demonstrates how to strategically use different sizes of PET aggregates to optimize the mechanical properties of ECC. The results highlight the potential of small- and medium-sized PET aggregates to balance strength and deformability, thereby enhancing the overall performance of ECC.

## 5. Conclusions

This study used PET aggregates of different particle sizes to replace natural aggregates in the preparation of PET-modified engineered cementitious composites (P-ECC). It investigated the performance of P-ECC from macro to micro levels, including workability, density, compressive strength, tensile strength, and microstructure. The study revealed the effects and mechanisms of PET aggregate particle size on P-ECC’s multiple cracking behavior, its tensile strain capacity, and the ITZ between the aggregate and the cement matrix. The following conclusions were drawn:

(1) The use of PET aggregates positively impacts the workability of ECC fresh paste, improving its processability and fiber dispersion. As the PET aggregate particle size increases, workability initially improves and then decreases. Among the samples, P-M showed the highest increase in workability, improving by 28.6% compared to the control group.
(2) An appropriate increase in PET aggregate particle size enhances P-ECC’s crack control ability, resulting in reduced crack width and increased crack density. Compared to P-S, the P-M samples showed a 20.1% reduction in average crack width and a 10.7% increase in crack density. However, when the PET aggregate particle size is too large (P-L), the crack control ability weakens due to stress concentration.
(3) Compared to ordinary ECC, the P-S and P-M samples showed improvements in tensile strength, ultimate tensile strain, and strain energy. P-S exhibited the highest increase in tensile strength and strain energy, with improvements of 18.1% and 92.8%, respectively. P-M contributed the highest increase in ultimate tensile strain, improving by 66.0%. In contrast, the P-L sample showed a significant decrease in tensile performance compared to the others.
(4) On the micro level, the ITZ between PET aggregates and the cement matrix is wider than that of traditional quartz powder, and the ITZ widens as the PET aggregate particle size increases. This characteristic introduces defects into the ECC, where appropriately sized defects enhance the tensile strain capacity of P-S and P-M, while excessively large defects significantly reduce the performance of P-L.

## Figures and Tables

**Table 1:** Chemical compositions of cementitious materials (unit: wt.%).

Cementitious Materials	CaO	SiO_2_	Al_2_O_3_	SO_3_	Fe_2_O_3_	MgO	TiO_2_	Other
Cement	67.8	16.5	3.94	4.55	4.51	1.04	0.26	1.4
FA	4.88	51.9	31.1	1.07	4.97	0.85	1.38	3.85

**Table 2:** Physical and mechanical properties of PE fiber.

Length (mm)	Diameter (μm)	Modulus of Elasticity (GPa)	Strength (MPa)	Density (g/cm^3^)	Elongation (%)
18	24	116	3000	0.97	3

**Table 3:** Physical and mechanical properties of QP and PET aggregates.

Aggregate Type	D50 (μm)	Modulus of Elasticity (GPa)	Density (g/cm^3^)	Elongation (%)
QP	120	76	2.68	-
PET-S	21	4	1.38	9.5
PET-M	107			
PET-L	244			

**Table 4:** Mix proportion of P-ECC (unit: kg/m^3^).

Group	Cement	FA	QP	PET Aggregate	Water	HRWRA	PE	VMA	DF
ECC	937	401	401	0	335	5.4	19.4	0.21	1.61
P-S			341	31.4					
P-M									
P-L									

**Table 5:** The density of P-ECC (unit: kg/m^3^).

Group	Wet Density (kg/m^3^)	Dry Density (kg/m^3^)	Density Difference (kg/m^3^)
ECC	2118	1940	178
P-S	2096	1921	175
P-M	2097	1918	179
P-L	2097	1913	184

**Table 6:** The compressive strength of the P-ECC samples.

Group	Compressive Strength (MPa)	Standard Deviation (MPa)	Relative Strength
ECC	98.2	2.0	1.00
P-S	89.9	1.7	0.92
P-M	89.2	0.9	0.91
P-L	81.5	1.3	0.83

**Table 7:** Summary of the crack parameters of the P-ECC for direct tensile testing.

Group	Average Crack Width (μm)	Crack Density (mm^−1^)
ECC	204 (11)	0.17 (0.02)
P-S	214 (7)	0.28 (0.03)
P-M	171 (4)	0.35 (0.04)
P-L	176 (9)	0.18 (0.02)

Note: The values of standard deviation are in parentheses.

**Table 8:** Summary of the tensile characteristic parameters of the P-ECC.

Group	Cracking Strength *σ*_tc_ (MPa)	Cracking Strain *ε*_tc_ (%)	Tensile Strength *σ*_t_ (MPa)	Ultimate Tensile Strain *ε*_t_ (%)
ECC	5.29 (1.16) a	0.24 (0.10) b	8.53 (0.12) c	3.71 (0.40) b
P-S	4.10 (0.64) ab	0.15 (0.08) b	10.07 (0.37) a	5.98 (0.63) a
P-M	3.37 (1.04) b	0.11 (0.06) b	9.12 (0.32) b	6.16 (0.88) a
P-L	3.42 (0.34) c	0.15 (0.02) a	7.97 (0.21) d	3.47 (1.07) b

Note: the values are standard deviations in parentheses. Values with the letters a, b, c, and d are significantly different across columns (*p* < 0.05).

**Table 9:** The strain energy of the P-ECC.

Group	Strain Energy, *g*_t_ (kJ/m^3^)	Standard Deviation (kJ/m^3^)	Relative Strength
ECC	263	54	1.00
P-S	507	85	1.93
P-M	485	105	1.84
P-L	233	79	0.89

## Data Availability

The data presented in this study are available on request from the corresponding author. The data are not publicly available due to confidentiality issues.

## References

[1] Xu L., Huang B., Li V.C., Dai J. (2022). High-strength high-ductility engineered/strain-hardening cementitious composites (ECC/SHCC) incorporating geopolymer fine aggregates. Cem. Concr. Compos..

[2] Li V.C., Bos F.P., Yu K., Mcgee W., Ng T.Y., Figueiredo S.C., Nefs K., Mechtcherine V., Nerella V.N., Pan J. (2020). On the emergence of 3d printable engineered, strain hardening cementitious composites (ECC/SHCC). Cem. Concr. Res..

[3] Li V.C. (2012). Tailoring ecc for special attributes: A review. Int. J. Concr. Struct. Mater..

[4] Lin J., Luo R., Su J., Guo Y., Chen W. (2024). Coarse synthetic fibers (PP and POM) as a replacement to steel fibers in uhpc: Tensile behavior, environmental and economic assessment. Constr. Build. Mater..

[5] Li V.C. (2019). Introduction to engineered cementitious composites (ECC). Engineered Cementitious Composites (ECC): Bendable Concrete for Sustainable and Resilient Infrastructure.

[6] Peng Y., Zheng D., Pan H., Yang J., Lin J., Lai H., Wu P.-Z., Zhu H.-Y. (2023). Strain hardening geopolymer composites with hybrid POM and UHMWPE fibers: Analysis of static mechanical properties, economic benefits, and environmental impact. J. Build. Eng..

[7] Huo Y., Liu T., Lu D., Han X., Sun H., Huang J., Ye X., Zhang C., Chen Z., Yang Y. (2023). Dynamic tensile properties of steel fiber reinforced polyethylene fiber-engineered/strain-hardening cementitious composites (PE-ECC/SHCC) at high strain rate. Cem. Concr. Compos..

[8] Wang Y., Liu F., Yu J., Dong F., Ye J. (2020). Effect of polyethylene fiber content on physical and mechanical properties of engineered cementitious composites. Constr. Build. Mater..

[9] Lin J., Liu R., Liu L., Zhuo K., Chen Z., Guo Y. (2024). High-strength and high-toughness alkali-activated composite materials: Optimizing mechanical properties through synergistic utilization of steel slag, ground granulated blast furnace slag, and fly ash. Constr. Build. Mater..

[10] Lin J., Song Y., Xie Z., Guo Y., Yuan B., Zeng J., Wei X. (2020). Static and dynamic mechanical behavior of engineered cementitious composites with PP and PVA fibers. J. Build. Eng..

[11] Zhu H., Xiong Z., Song Y., Zhou K., Su Y. (2024). Effect of expansion agent and glass fiber on the dynamic splitting tensile properties of seawater–sea-sand concrete. Bulidings.

[12] Li S., Chen X., Liu Z., Lu Y., Wang H. (2023). Axial behavior of pre-damaged RC columns strengthened with CFRP textile grid-reinforced ECC matrix composites. J. Build. Eng..

[13] Lyu B., Ding C., Guo L., Chen B., Wang A. (2021). Basic performances and potential research problems of strain hardening geopolymer composites: A critical review. Constr. Build. Mater..

[14] Wang S., Lin C., Li S., Lu Y. (2024). Compressive performance and analytical modeling of early strength seawater sea sand engineered cementitious composites. J. Build. Eng..

[15] Zheng Y., Zhang L.F., Xia L.P. (2018). Investigation of the behaviour of flexible and ductile ECC link slab reinforced with FRP. Constr. Build. Mater..

[16] Wu X., Tian J., Ma H., Zheng Y., Hu S., Wang W., Du Y., Huang W., Sun C., Zhu Z. (2020). Investigation on interface fracture properties and nonlinear fracture model between ecc and concrete subjected to salt freeze-thaw cycles. Constr. Build. Mater..

[17] Yang Z., Pan H., Jiang Z., Lv J., Ruan G., Lai H., Lin J.-X. (2024). Pseudo strain-hardening alkali-activated composites with up to 100 % rubber aggregate: Static mechanical properties analysis and constitutive model development. Constr. Build. Mater..

[18] Chen G., Zheng D., Chen Y., Lin J., Lao W., Guo Y., Chen Z.-B., Lan X.-W. (2023). Development of high performance geopolymer concrete with waste rubber and recycle steel fiber: A study on compressive behavior, carbon emissions and economical performance. Constr. Build. Mater..

[19] Guo Y.C., Zhang J.H., Chen G., Chen G.M., Xie Z.H. (2014). Fracture behaviors of a new steel fiber reinforced recycled aggregate concrete with crumb rubber. Constr. Build. Mater..

[20] Huang B., Li Q., Xu S., Zhang L. (2019). Static and fatigue performance of reinforced concrete beam strengthened with strain-hardening fiber-reinforced cementitious composite. Eng. Struct..

[21] Li V.C., Leung C.K.Y. (1992). Steady-state and multiple cracking of short random fiber composites. J. Eng. Mech..

[22] Xu L., Huang B., Dai J. (2021). Development of engineered cementitious composites (ECC) using artificial fine aggregates. Constr. Build. Mater..

[23] Lyu B., Wang A., Zhang Z., Liu K., Xu H., Shi L., Sun D. (2019). Coral aggregate concrete: Numerical description of physical, chemical and morphological properties of coral aggregate. Cem. Concr. Compos..

[24] Chen Z., Pan H., Zhuo K., Su J., Xie B., Lin J., Guo Y. (2024). Dynamic compressive behavior of environmentally friendly high-strength concrete: Experimental investigation and modelling. Constr. Build. Mater..

[25] Chen W., Wang Q., Huang Z., Du H. (2024). Efficient utilization of waste marine clay for fine aggregate to develop sustainable and cost-effective strain-hardening cement-based composites. Constr. Build. Mater..

[26] Zhou Y., Gong G., Huang Y., Chen C., Huang D., Chen Z., Guo M. (2021). Feasibility of incorporating recycled fine aggregate in high performance green lightweight engineered cementitious composites. J. Clean Prod..

[27] Chandrasekhar C.R.N. (2023). GDR. Engineered cementitious composites (ECC) with manufactured sand (M-sand) for pavement applications. Compos. Commun..

[28] Xia D., Chen R., Zhang D., Cheng J. (2022). Relationship between fractal dimension and properties of engineered cementitious composites with different aggregates. Materials.

[29] Guan X., Li Y., Liu T., Zhang C., Li H., Ou J. (2019). An economical ultra-high ductile engineered cementitious composite with large amount of coarse river sand. Constr. Build. Mater..

[30] Qiu X., Chen W., Li L., Li H., Liu H. (2023). The effects of particle sizes of expanded perlite on the mechanical properties and chloride penetration resistance of ECCS. J. Build. Eng..

[31] Ma J., Hesp S.A.M. (2022). Effect of recycled polyethylene terephthalate (PET) fiber on the fracture resistance of asphalt mixtures. Constr. Build. Mater..

[32] Smaoui H., Trabelsi A., Kammoun Z., Aouicha B. (2023). Mechanical, physical, blast waves and ballistic impact resistance properties of a concrete incorporating thermally treated pet inclusions. Constr. Build. Mater..

[33] Ali B.T.I., Widiastuti N., Kusumawati Y., Jaafar J. (2023). Utilization of polyethylene terephthalate (PET) plastic bottle waste as membrane with several modifications for the removal of chromium ions in wastewater. Mater. Today Proc..

[34] Pu M., Zhou X., Liu X., Fang C., Wang D. (2023). A facile, alternative and sustainable feedstock for transparent polyurethane elastomers from chemical recycling waste pet in high-efficient way. Waste Manag..

[35] Chen H., Zuo Z., Tian Q., Xue S., Qiu F., Peng X., Zhang T. (2023). Waste to treasure: A superwetting fiber membrane from waste pet plastic for water-in-oil emulsion separation. J. Clean. Prod..

[36] Resende D.M., Mendes V.F., Carvalho V.R., Nogueira M.A., de Carvalho J.M.F., Peixoto R.A.F. (2024). Coating mortars produced with recycled pet aggregates: A technical, environmental, and socioeconomic approach applied to Brazilian social housing. J. Build. Eng..

[37] Almeshal I., Tayeh B.A., Alyousef R., Alabduljabbar H., Mustafa Mohamed A., Alaskar A. (2020). Use of recycled plastic as fine aggregate in cementitious composites: A review. Constr. Build. Mater..

[38] Dai P., Lyu Q., Zong M., Zhu P. (2024). Effect of waste plastic fibers on the printability and mechanical properties of 3d-printed cement mortar. J. Build. Eng..

[39] Saikia N., de Brito J. (2014). Mechanical properties and abrasion behaviour of concrete containing shredded PET bottle waste as a partial substitution of natural aggregate. Constr. Build. Mater..

[40] Akçaözoğlu S., Atiş C.D., Akçaözoğlu K. (2010). An investigation on the use of shredded waste pet bottles as aggregate in lightweight concrete. Waste Manag..

[41] Chan K., Zinchenko A. (2023). Design and synthesis of functional materials by chemical recycling of waste polyethylene terephthalate (PET) plastic: Opportunities and challenges. J. Clean. Prod..

[42] Alqahtani F.K., Ghataora G., Dirar S., Khan M.I., Zafar I. (2018). Experimental study to investigate the engineering and durability performance of concrete using synthetic aggregates. Constr. Build. Mater..

[43] Vinod Kumar R., Rupesh Kumar D.D. (2023). Recycled plastic (HDPE) coarse aggregate manufacturing method and performance in concrete. Mater. Today Proc..

[44] Frigione M. (2010). Recycling of pet bottles as fine aggregate in concrete. Waste Manag..

[45] Kangavar M.E., Lokuge W., Manalo A., Karunasena W., Ozbakkaloglu T. (2023). Development of sustainable concrete using recycled polyethylene terephthalate (PET) granules as fine aggregate. Dev. Built Environ..

[46] Zhen H., Xiong Z., Song Y., Li L., Qiu Y., Zou X., Chen B., Chen D., Liu F., Ji Y. (2024). Early mechanical performance of glass fibre-reinforced manufactured sand concrete. J. Build. Eng..

[47] Xue H., Zhu H., Guo M., Shao S., Zhang S., Zhang Y. (2023). Modeling for predicting triaxial mechanical properties of recycled aggregate concrete considering the recycled aggregate replacement. Constr. Build. Mater..

[48] Wang A., Zheng Y., Zhang Z., Liu K., Li Y., Shi L., Sun D. (2020). The durability of alkali-activated materials in comparison with ordinary portland cements and concretes: A review. Engineering.

[49] Dobiszewska M., Bagcal O., Beycioğlu A., Goulias D., Köksal F., Płomiński B., Ürünveren H. (2023). Utilization of rock dust as cement replacement in cement composites: An alternative approach to sustainable mortar and concrete productions. J. Build. Eng..

[50] Wang A., Lyu B., Zhu Y., Liu K., Guo L., Sun D. (2021). A gentle acid-wash and pre-coating treatment of coral aggregate to manufacture high-strength geopolymer concrete. Constr. Build. Mater..

[51] Janardhan P., Narayana H., Darshan N. (2023). Compressive strength studies of concrete with partial replacement of cement and fine aggregate with incinerated solid waste and recycled plastic waste. Mater. Today Proc..

[52] Chanda A., Rawat S., Thakkar S., Dave U., Joshi T. (2023). Influence on compressive strength of concrete by partial replacement of river sand with fly ash geopolymer sand. Mater. Today Proc..

[53] Guo Y., Li X., Zhang J., Lin J. (2023). A review on the influence of recycled plastic aggregate on the engineering properties of concrete. J. Build. Eng..

[54] Reis J.M.L., Carneiro E.P. (2012). Evaluation of pet waste aggregates in polymer mortars. Constr. Build. Mater..

[55] Nikbin I.M., Dezhampanah S., Charkhtab S., Mehdipour S., Shahvareh I., Ebrahimi M., Pournasir A., Pourghorban H. (2022). Life cycle assessment and mechanical properties of high strength steel fiber reinforced concrete containing waste pet bottle. Constr. Build. Mater..

[56] Marzouk O.Y., Dheilly R.M., Queneudec M. (2007). Valorization of post-consumer waste plastic in cementitious concrete composites. Waste Manag..

[57] Fakhruddin Irmawaty R., Djamaluddin R. (2022). Flexural behavior of monolith and hybrid concrete beams produced through the partial replacement of coarse aggregate with pet waste. Structures.

[58] Kangavar M.E., Lokuge W., Manalo A., Karunasena W., Frigione M. (2022). Investigation on the properties of concrete with recycled polyethylene terephthalate (PET) granules as fine aggregate replacement. Case Stud. Constr. Mater..

[59] Ahmed M.F. (2024). Assessment of waste polyethylene terephthalate (PET) as sand in sustainable geopolymer concrete: Non-destructive tests investigation. Proceedings of the 7th International Conference on Civil Engineering.

[60] Kaur G., Pavia S. (2020). Physical properties and microstructure of plastic aggregate mortars made with acrylonitrile-butadiene-styrene (ABS), polycarbonate (PC), polyoxymethylene (POM) and ABS/PC blend waste. J. Build. Eng..

[61] (2020). Standard Test Method for Flow of Hydraulic Cement Mortar.

[62] (2023). Density (Unit Weight), Yield, and Air Content (Gravimetric) of Concrete.

[63] (2021). Standard Test Method for Compressive Strength of Hydraulic Cement Mortars (using 2-in. Or [50 mm] Cube Specimens).

[64] Yokota H., Rokugo K., Sakata N. (2007). JSCE Recommendations for design and construction of high performance fiber reinforced cement composite with multiple fine cracks. High Performance Fiber Reinforced Cement Composites.

[65] Li V.C. (2019). Processing of engineered cementitious composites (ECC). Engineered Cementitious Composites (ECC): Bendable Concrete for Sustainable and Resilient Infrastructure.

[66] Saikia N., de Brito J. (2012). Use of plastic waste as aggregate in cement mortar and concrete preparation: A review. Constr. Build. Mater..

[67] Babu K.G., Babu D.S. (2003). Behaviour of lightweight expanded polystyrene concrete containing silica fume. Cem. Concr. Res..

[68] (2019). Technical Standard for Application of Lightweight Aggregate Concrete.

[69] Kunthawatwong R., Sylisomchanh L., Pangdaeng S., Wongsa A., Sata V., Sukontasukkul P., Chindaprasirt P. (2022). Recycled non-biodegradable polyethylene terephthalate waste as fine aggregate in fly ash geopolymer and cement mortars. Constr. Build. Mater..

[70] Li V.C. (2019). Mechanical properties of engineered cementitious composites (ECC). Engineered Cementitious Composites (ECC): Bendable Concrete for Sustainable and Resilient Infrastructure.

